# Cutting-edge Treatment for Gynecological Malignancies

**DOI:** 10.14789/jmj.JMJ22-0044-R

**Published:** 2023-03-29

**Authors:** YASUHISA TERAO

**Affiliations:** 1 Department of Obstetrics and Gynecology, Faculty of Medicine, Juntendo University, Tokyo, Japan; 1 Department of Obstetrics and Gynecology, Faculty of Medicine, Juntendo University, Tokyo, Japan

**Keywords:** gynecological surgery, cervical cancer, endometrial cancer, ovarian cancer, minimal invasive surgery

## Abstract

Gynecological malignant tumors can develop in the vulva, vagina, uterus, fallopian tubes, or ovaries in the female reproductive tract. The cervix, uterine body, and ovaries are particularly common sites for malignant tumors. Surgery, radiation, and drug therapy are the main treatment modalities for gynecological cancers, with surgery being the most important of them.

We started laparoscopic surgery for uterine endometrial cancer as an advanced medical treatment in 2011 and contributed to its insurance coverage. We were able to reproduce our laparoscopic surgery more easily using the da Vinci Xi system for robotic surgery. We have now switched from laparoscopic surgery for endometrial cancer to robotic surgery and have been able to perform them safely and reliably.

In the case of cervical cancer, the results of the Laparoscopic Approach to Cervical Cancer (LACC) trial, which compared the prognosis of two groups of radical hysterectomy for early-stage cervical cancer: conventional open surgery and laparoscopic/robotic (minimally invasive) surgery, showed that minimally invasive surgery resulted in more pelvic recurrences and had a worse prognosis compared with open surgery. The trend toward minimally invasive surgery for cervical cancer has stagnated worldwide.

Ovarian cancer has few symptoms in the early stages and is often found at stage III or IV, when the cancer has spread throughout the abdominal cavity. As residual tumor after surgery correlates with prognosis in ovarian cancer, debulking surgery should be performed to achieve complete resection. Therefore, peritoneal or bowel resection is often required to remove disseminated or metastatic tumors. We also performed prophylactic salpingo-oophorectomy to prevent ovarian and fallopian tube cancers in patients with *BRCA1/2* gene variants.

The uterus and ovaries are organs necessary for pregnancy and childbirth, and cancer of the uterus or ovaries in women of childbearing age may result in infertility. Surgery and adjuvant treatment may affect marriage, childbirth, and sexual life; therefore, it is important to ensure the cure of cancer and to provide patients with treatment methods that allow them to live their lives as women.

## Introduction

Gynecological cancers are specific to women, and malignant tumors can develop in the vulva, vagina, uterus, fallopian tubes, and ovaries in the female reproductive tract, but the cervix, uterine body, and ovaries are particularly common sites. Surgery, radiation, and drug therapy are the main treatment modalities for gynecological cancers, with surgery being the most important treatment modality.

With advances in optical equipment and power sources, laparoscopic surgery is now indicated for malignant cases that were once considered difficult, and laparoscopic surgery for endometrial and cervical cancers have been covered by insurance since 2014 and 2018. In addition, robotic surgery for uterine endometrial cancer was covered by insurance in 2018. Robotic surgery for gynecological diseases is more common than laparoscopic surgery in the United States^1)^. In our hospital, we have been actively performing laparoscopic and robotic surgeries, and we would like to introduce them here. In ovarian cancer, postoperative residual tumor correlates with prognosis, so debulking surgery should be performed with the aim of achieving complete surgery with no visible residual tumor. Therefore, peritoneal and bowel resection are often required to remove disseminated or metastatic tumors as much as possible, which is not minimally invasive. In our department, we perform complete resection of ovarian cancer without complications, in cooperation with the departments of colorectal surgery, hepatobiliary surgery, and urology. We also introduce the latest surgical treatments for gynecological cancer, such as prophylactic salpingo-oophorectomy, to prevent ovarian and fallopian tube cancers in patients with *BRCA1/2* gene variants.

## Uterine endometrial cancer

Endometrial cancer is a cancer that develops in the endometrium of the uterus. The occurrence site and cause are different from those of cervical cancer of the uterus. The number of cases of endometrial cancer began to increase in the late 40s around the time of menopause and is most common in women in their 50s and 60s. As it is difficult to accurately diagnose endometrial cancer, which is found in the body of the uterus, surgery is performed after estimating the clinical stage based on preoperative imaging, and the pathological stage is classified again after surgery. The risk of recurrence was assessed after surgery. Based on the stage and risk of recurrence, it is decided whether adjuvant treatment is necessary after surgery. Surgery is the first choice of treatment for endometrial cancer, regardless of the advanced stage. However, the type of surgery performed depends on its stage. If the risk of surgery is high owing to advanced age, serious diabetes, heart disease, severe obesity, or if the cancer has already spread throughout the body and surgery is not expected to be effective, chemotherapy, radiation therapy, or palliative care may be used.

In stage I cases, in which the cancer is presumed to be confined to the uterine body only, the standard procedure is a total hysterectomy, in which the entire uterus is removed, and a bilateral salpingo-oophorectomy, in which both the ovaries and fallopian tubes are removed. Depending on the histological type and degree of invasion into the myometrium, pelvic or paraaortic lymph nodes may be removed.

Until 2010, all cases of endometrial cancer were treated with open surgery in our hospital. The abdominal incision was a midline incision at the level of the umbilicus or up to the xiphoid process. This is a highly invasive surgery. At that time, there was little evidence of minimally invasive surgery (MIS) for gynecological malignancies in Japan. We started laparoscopic surgery for early-stage endometrial cancer because we thought that if we could reproduce the open surgical technique using laparoscopy, we could establish a standard procedure that was safe and curative while maintaining surgical completion and accuracy. The indications for laparoscopic surgery were G1 or G2 endometrioid carcinoma with myometrial invasion ≤1/2, and the basic surgical techniques were total hysterectomy, bilateral salpingo-oophorectomy, and pelvic lymphadenectomy. The instruments used were standardized. To prevent tumor dispersal, a uterine manipulator was not inserted, and the uterus and lymph nodes were removed from the body in a bag. The results showed that laparoscopic surgery prolonged the operation time compared with open surgery but decreased the amount of blood loss, hospital stay, and visual analogue scale score on postoperative day 1. There were no significant differences in progression free survival and overall survival^2)^. The results contributed to the insurance coverage of laparoscopic surgery for uterine endometrial cancer.

However, laparoscopic surgery requires more time to master than open surgery. This is because it is a magnified view, and it is necessary to visualize the three-dimensional (3D) in a two-dimensional view. In addition, eye-hand coordination is required. In robotic surgery, once surgeons are accustomed to the magnified vision, robotic surgery provides 3D vision, and forceps can be moved freely like in open surgery^3, 4)^.

Table 1 shows a comparison of short- and long-term outcomes, invasiveness, learning curve, cost effectiveness, and easy accessibility of open, laparoscopic, and robotic surgery in early endometrial cancer surgery^5-9)^. Easy accessibility was further improved by using the Da Vinci Xi system. In 2018, robotic surgery for uterine cancer was covered by the insurance. Eighty-two cases (41 robotic and 41 laparoscopic surgeries) of total hysterectomy and bilateral adnexectomy with MIS for uterine cancer between 2019 and 2020 were reviewed. Robotic surgery had similar blood loss and complication rates as those of laparoscopic surgery. Robotic surgery shortened the operating time compared to laparoscopic surgery. Our laparoscopic surgery for endometrial cancer can be more easily reproduced using the da Vinci Xi system. We have now switched all laparoscopic surgeries for endometrial cancer to robotic surgery and have been able to perform them safely and reliably.

**Table 1:** Benefit for minimally invasive surgery in endometrial cancer

	Open surgery	Laparoscopic surgery	Robotic surgery
Short term outcome	〇	〇	〇
Long-term outcome	〇	〇	〇
Less invasiveness	△	〇	〇
Learning curve	〇	△	〇
Cost effectiveness	△	〇	△
Easy accessibility	〇	〇	〇

The meaning of 〇 is equivalent to the others, △ means inferior to others

## Cervical cancer

Cervical cancer occurs in the cervix, which is the entrance point to the uterus. Cancer develops near the junction of squamous and columnar cells that line the cervical surface. It is mainly classified as a squamous cell carcinoma or adenocarcinoma. Cervical cancer is most common in women in their late 30s and 40s. It is caused by human papillomavirus (HPV). Persistent infection with highly carcinogenic HPV causes dysplasia (precancerous lesions), and some of these lesions become cancerous over a period of 5-10 years, progressing from mild-to-moderate to severe dysplasia. In the case of mild or moderate dysplasia, the patient is followed-up regularly, and in the case of severe dysplasia or carcinoma in situ, cervical conization is performed. When further carcinogenesis develops, cervical cancer is classified into four stages: stages I, II, III, and IV. Cervical conization that can preserve fertility can be performed if the cancer is carcinoma in situ, in which the cancer remains in the epithelial cells, and microinvasive carcinoma (stage IA), in which the cancer invades the stroma on a millimeter scale.

Stage IA to IIb cervical cancer is operable. However, depending on the stage, there are several methods for uterine removal. Radical hysterectomy is one of the most difficult gynecological surgeries. MIS was performed for both cervical and endometrial cancer. However, the LACC trial published in 2018 changed the trend toward MIS. In a 13-country comparative study, the authors compared the outcomes of two groups of radical hysterectomies for early-stage cervical cancer: conventional laparotomy (open) and laparoscopic/robotic surgery (MIS). The results showed that MIS resulted in greater pelvic recurrence and worse prognosis than open surgery^10)^. In a retrospective study, MIS had a poorer prognosis than open surgery^11, 12)^. The trend toward MIS for cervical cancer has been stalled worldwide.

In the case of local recurrence after radiotherapy for cervical cancer, surgical therapy is now the treatment of choice if there are no distant or lymph node metastases. In such cases, total pelvic exenteration (TPE) or anterior or posterior segment excision is a radical surgical procedure that removes organs, including the bladder, urethra, rectum, anus, vagina, uterus, fallopian tubes, ovaries, and vulva. TPE has become safer than in the past, with a mortality rate of approximately 0.5 to 2%^13-15)^. However, postoperative complications in gynecology are as high as 67%^15)^.

Four TPEs were performed at our hospital between 2010 and 2021. The median age of the patients was 59 years. The indicated diseases were cervical cancer recurrence (all after concurrent chemoradiotherapy) in three patients and first vaginal sarcoma in one patient. The mean operative time was 667±80 min, and the mean blood loss was 3,216±2,820 mL. All patients underwent laparotomy and transperineal surgery, which was performed jointly with the Department of Colorectal Surgery and the Department of Urology. All patients required blood transfusions, and the only complication of grade III or higher according to the Clavien-Dindo classification was grade IIIa bowel obstruction. The mean length of hospital stay was 31 (±7) days. All patients had negative surgical margins, three were disease-free, and one was alive. As a surgical innovation, we previously operated safely on a giant myoma by using a balloon catheter to block the blood flow^16)^. As an application of this technique, intraaortic balloon occlusion was used to safely perform the surgery. However, even without serious complications, mental acceptance of double stoma requires time; therefore, it is essential to provide mental care from multiple professions from an early stage.

## Ovarian cancer

Ovarian cancer has few symptoms in the early stages. The organs below the diaphragm, including the uterus, ovaries, stomach, and intestines, are covered by peritoneum. Ovarian cancer is similar to cancers arising in the fallopian tubes and peritoneum; therefore, these cancers are treated together as same. Various tumors can occur in the ovaries. Depending on the tumor location, they are classified into three main categories: (1) epithelial tumors, (2) sex cord stromal tumors, and (3) germ cell tumors. Tumors are further divided into (1) benign, (2) borderline malignant, and (3) malignant. Epithelial tumors are the most common type of ovarian tumors, accounting for approximately 90% of all malignant ovarian tumors. Generally, the term ovarian cancer refers to malignant epithelial tumors.

Ovarian cancer is one of the most difficult cancers to detect early due to a lack of symptoms and appropriate screening methods. More than 40% of ovarian cancers are found in advanced stages III and IV.

**Figure 1:**
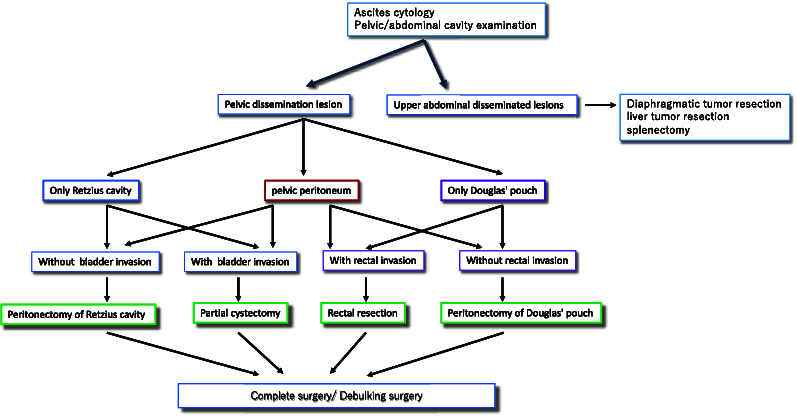
Strategy for ovarian cancer surgery Observe the peritoneal cavity after collecting ascites. If the tumor is in the upper abdomen, it is evaluated whether it can be completely removed. If the tumor is only in the pelvis, it can be completely removed following the procedure.

If ovarian cancer is suspected, surgery is often the first step at an advanced stage. Intraoperative pathology will determine whether an ovarian tumor is benign or malignant. In the case of borderline malignancy or malignancy, both ovaries, uterus, and omentum are removed. If the cancer is scattered in the abdomen at the time of surgery (peritoneal dissemination), a tumor resection surgery is performed to remove as much of the cancer as possible. For ovarian cancer, the less tumor remains after surgery, the better is the prognosis^17)^. Therefore, surgery should be performed with maximum debulking with the goal of achieving a complete surgery, in which there is no gross residual tumor. Peritoneal or partial bowel resection is often required to remove disseminated or metastatic lesions. In our hospital, the percentage of patients with ovarian cancer who underwent intestinal resection was 7.1% (43 cases) between 1995-2011 and 57 cases (10.7%) between 2012-2020, and the percentage has been increasing gradually. The surgical strategy for ovarian cancer with pelvic peritoneal dissemination at our hospital is shown in Figure 1. The abdominal cavity was observed after laparotomy. We evaluated whether all the tumors could be removed. If the cancer is not resected, chemotherapy should be administered followed by interval debulking surgery. If only pelvic peritoneal dissemination was present, it could be removed. If bladder or intestinal tract invasion is observed, partial resection of the bladder, sigmoid colon, or rectum is performed. If there is no involvement of the bladder or intestinal tract, the peritoneum is resected. Usually, leakage of anastomosis occurs in 2.8-30% of the cases, 75% of which occur at the rectal anastomosis site^18)^. The rate of anastomotic leakage in ovarian cancer is 1%. Compared to colorectal cancer surgery, there is no need to cut the root of the blood vessel; therefore, the complication rate is low. We performed complete resections safely and without complications, with the cooperation of the departments of colorectal surgery, hepatobiliary surgery, and urology.

Genetic factors are strongly associated with approximately 10% of the ovarian cancers, and pathological variants of *BRCA1* or *BRCA2* predispose ovarian and breast cancers. Risk-reducing salpingo-oophorectomy reduces the risk of developing ovarian cancer and the overall mortality in women with *BRCA 1/2* genetic variants^19-21)^ and is recommended in the guidelines.

## Conclusion

Surgery is one of the most important treatments for gynecological cancers. Surgery is the most efficient way to remove cancer cells; however, it is also invasive. The choice of surgery should be based on patient's age and general condition. In addition, the uterus and ovaries are organs necessary for pregnancy and childbirth, and cancers of the uterus or ovaries in childbearing age may result in loss of fertility. It is important to perform surgery that preserves the possibility of fertility. Surgery and subsequent treatment may affect marriage, childbirth, and sex life; therefore, it is important to provide patients with treatment methods that allow them to face their “womanhood” while ensuring the cure of cancer.

## Funding

No funding was received.

## Author contributions

YT wrote and approved the final manuscript.

## Conflicts of interest statement

The author declares that there are no conflicts of interest.
